# Effects of mesoscale eddies on intraseasonal variability of intermediate water east of Taiwan

**DOI:** 10.1038/s41598-022-13274-2

**Published:** 2022-06-02

**Authors:** Qiang Ren, Fei Yu, Feng Nan, Yuanlong Li, Jianfeng Wang, Yansong Liu, Zifei Chen

**Affiliations:** 1 Key Laboratory of Ocean Circulation and Waves, Institute of Oceanology, Chinese Academy of Sciences, Qingdao, China; 2 Center for Ocean Mega-Science, Chinese Academy of Sciences, Qingdao, 266071 China; 3 Pilot National Laboratory for Marine Science and Technology (Qingdao), Qingdao, 266071 China

**Keywords:** Ocean sciences, Physical oceanography

## Abstract

The intraseasonal variability of the intermediate water (IW) and its characteristics east of Taiwan are studied utilizing 17 months of long-term, continuous and synchronous measurements of temperature, salinity and current from mooring sites deployed at 122° E/23° N from January 2016 to May 2017. Direct measurements revealed water masses east of Taiwan alternately show complete South China Sea Intermediate Water or North Pacific Intermediate Water (NPIW) characteristics, mostly in a mixed state, with NPIW dominating 70% of the time. For the first time, it is demonstrated that the variation of IW with periods of ~ 80 days is mainly related to mesoscale eddies. Anticyclonic (cyclonic) eddies corresponding to an increase (decrease) in temperature and salinity in the intermediate layer. Further mechanism analysis indicates the vertical motion of the water mass inside the eddies is one of the reasons for the thermohaline change in the intermedaiter layer. In addition, the anticyclonic eddies may increase the salinity concentration gradient across the Luzon Strait, and the enhanced advection is favorable to the outflow of water masses in the South China Sea. When the cyclonic eddies acts on the eastern part of Taiwan, the influence of the northward advection is weakened and the southward flow on its left side is more favorable to the transport of NPIW.

## Introduction

The Kuroshio is a strong western boundary current in the North Pacific Ocean that originates from the North Equatorial Current. It brings heat and salt from low latitudes to mid and high latitudes and has an important impact on air-sea interactions and climate change along its path^1–3^. Therefore, understanding the characteristics of the Kuroshio water masses is of great significance for studying its poleward volume, heat and salinity transport and for making future climate predictions.

**Figure 1:**
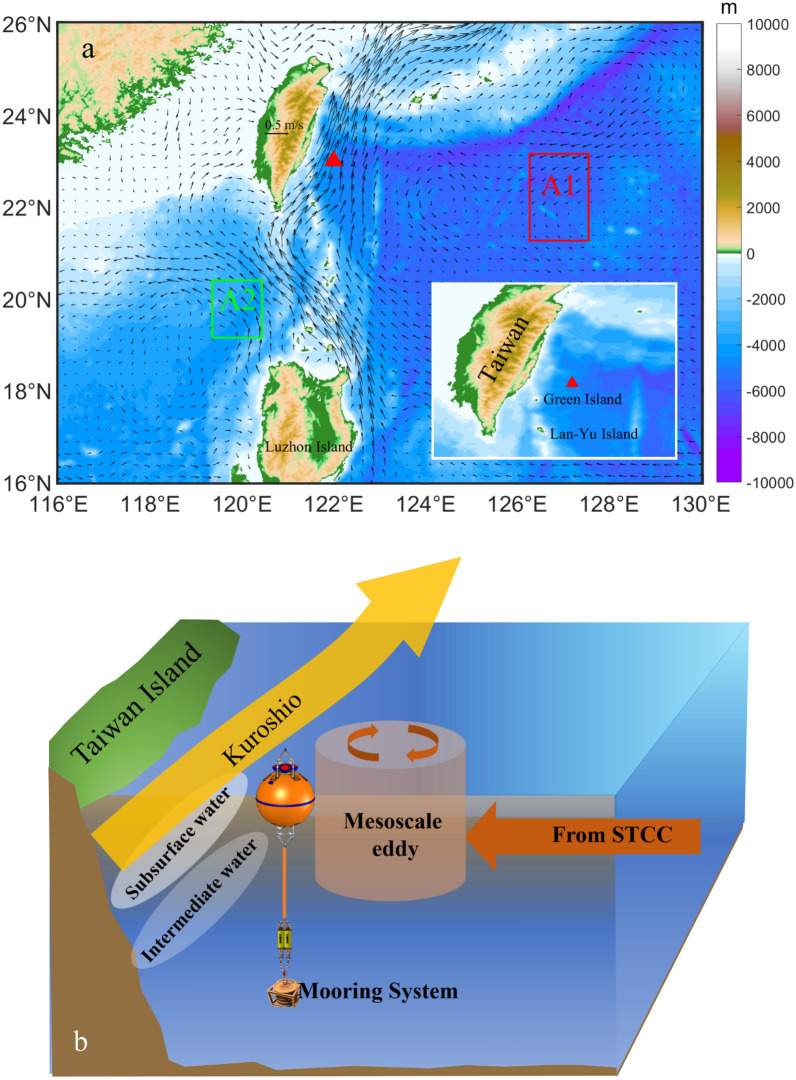
The surface geostrophic currents and a schematic diagram of the water mass distribution around the eastern Taiwan. (**a**) Isobaths (in meters) from data according to the ETOPO1 Global Relief Model (color shading) and average surface geotropic currents between Jan 2016 and May 2017 from AVISO data (vectors). For analysis of properties of IW in this study, the average characteristics of the water masses in two typical regions were selected to represent North Pacific Intermediate Water (in the red A1 box) and South China Sea Intermediate Water (in the green A2 box). The map at the lower right corner of (**a**) shows the zoomed-in topography east of Taiwan. (**b**) Schematic diagram of the water mass distribution and circulation east of Taiwan. The mooring system was deployed from Jan 2016 to May 2017. The topographic data from following website: https://www.ngdc.noaa.gov/mgg/global/etopo1sources.html. Figures were plotted using MATLAB R2016b (http://www.mathworks.com/).

In the Northwest Pacific Ocean, the circulation background is complex, water masses are mainly divided into subsurface water masses and intermediate water masses (Fig. 1b), and each has distinct characteristics. Intermediate water is widely distributed in the North Pacific and cold with relatively low salinity at depths of 400 ~ 800 m; it has a minimum salinity of approximately 34.10–34.20 psu and a potential density of 26.8 σ_θ_, which generally refers to North Pacific Intermediate Water (NPIW)^4,5^. The NPIW primarily originates in the Okhotsk Sea and is transported via the Oyashio current along Kuril Island and east of the Japanese island of Hokkaido^6–10^. Then, NPIW spreads throughout the North Pacific and can extend southward to ~ 15°N by the subtropical gyre; it has even been found in the Halmahera eddy (HE)^11^. At the same time, NPIW is transported through the Luzon Strait to the South China Sea where it forms South China Sea Intermediate Water (SCSIW) with a salinity minimum of approximately 34.4 psu at core depths of approximately 500 m^12–14^. In addition, many studies defined another water mass, referred to as Kuroshio Intermediate Water (KIW), along the western boundary from Luzon Island to an area east of Taiwan, according to plots of the average temperature versus salinity (${\uptheta }$-S) of the Kuroshio^7,15–17^. However, these papers report different sources of KIW. Mensah et al.^7^ claim that KIW is mostly found in the West Philippine Sea Basin along the Kuroshio, with saltier variations in NPIW. According to Chern and Wang^16^, Chen^15^, and Nakamura et al.^17^, KIW may contain both SCSIW and NPIW and is mostly found east of Taiwan.

During ongoing research on intermediate water east of Taiwan, many discussions concern the presence of SCSIW east of Taiwan. In previous work, SCSIW with a salinity of approximately 34.4 psu was found on the continental shelf northeast of Taiwan^16,18^. In a survey of the Luzon Strait and the Okinawa Trough, Chen^15^ also found evidence of SCSIW spreading to southern Japan though water east of Taiwan. Nakamura et al.^17^ used climatological data to describe IW east of Taiwan as a mixture of SCSIW and NPIW. Recently, Mensah et al.^6^ used temperature data at 580 m to develop an empirical formula to estimate IW salinity,they found that IW east of Taiwan contains SCSIW and NPIW. Mensah et al.^6^ also showed that IW salinity correlated with thickness of the Kuroshio. However, Chern and Wang^16^ did not identify SCSIW in the water north of Green Island, according to survey data from several hydrographic sections in the water east of Taiwan, and they claimed that the ridge between Taiwan and Green Island (less than 500 m deep) blocks the northward flow of water from the northern part of the South China Sea, and thus, prevents SCSIW from being carried into the area east of Taiwan. These findings suggest that intermediate waters along the Kuroshio east of Taiwan are highly variable, and researchers speculate that SCSIW and NPIW may alternate below the core of the Kuroshio.

Actually, most of the studies of IW in the area east of Taiwan use only a small amount of sectional data acquired during surveys and provide characteristics at a given point in time. As an area with a high incidence of mesoscale eddies propagating westward from the STCC, the Kuroshio east of Taiwan is affected by mesoscale eddies and experiences intraseasonal variability with periods of 80 ~ 100 d^3,19–27^. Due to the lack of simultaneous long-term continuous observations of the temperature, salinity and currents in this area, knowledge of the properties of IW east of Taiwan is remain limited, and this knowledge is not sufficient to clearly reveal the distribution characteristics and variations of IW in this area. For the first time, it has been possible to study the variations and processes of IW east of Taiwan using 17 months of simultaneous and continuous measurements of temperature, salinity and current. A subsurface mooring was deployed at 23° N, 122° E (red triangle in Fig. 1) from January 2016 to May 2017 to monitor the temperature, salinity and current upper 800 m. Although Mensah et al.^6^ hypothesized in his paper that the variation of IW are related to mesoscale eddies, whether IW in the intermediate layer is influenced by mesoscale eddies needs to be determined by direct measurement data, more than that the variation characteristics of IW and the relationship between IW and the Kuroshio are also not clearly.

## Data

### Mooring system data

The Institute of Oceanography, Chinese Academy of Sciences (IOCAS), conducted a large-scale survey of the Western Pacific in January 2016. During this survey, we deployed a subsurface mooring east of Taiwan at a water depth of 4900 m; in May 2017, we successfully recovered the mooring system using the research vessel R/V *Science* (location: 122° E, 23° N, Fig. 1). Figure S1 shows a simplified schematic diagram of the configuration and deployment of the subsurface mooring buoy system. The main floating ball integrated two up-looking and down-looking 75 kHz Acoustic Doppler Current Profilers (ADCPs) manufactured by Teledyne RD Instruments (TRDI), designed for use at depths of approximately 400 m, to measure currents at depths above 800 m according to the following parameters: the measurement interval was 1 h, the number of depth cells was 74, the bin size was 8 m, and the number of pings was 30 per measurement. At depths of 400–1000 m, we used conductivity-temperature-depth meters (CTDs, type: SBE37, manufactured by Sea Bird Instrument) at intervals of 100 m, and the sampling interval was 10 min. The current data from the ADCPs were controlled for quality, including a good threshold of 70% and a cutoff of 2 m/s for current speed. To filter out the influence of high-frequency signals, all current and CTD data were averaged daily for this study.

### AVISO altimetry and salinity data

An Archiving, Validation, and Interpretation of Satellite Data in Oceanography (AVISO) altimetry dataset was used in this paper. The sea level anomaly (SLA) and geostrophic current data were obtained from the commercial AVISO Global ARMOR3D L4 Reprocessed dataset (http://marine.copernicus.eu/services-portfolio/access-to-products/). The SLA data had spatial resolution of 1/4° × 1/4°, and the dataset extended approximately 17 months, from January 2016 to June 2017.

To analyze the temperature and salinity changes of IW, we selected the commercial data of Global Ocean Multi Observation Products, which is based on Global Ocean Observations (GOOPs). This commercial dataset is based on global ocean observations, using sea surface temperature (SST), sea level abnormalities (SLAs), average dynamic terrain (ADT) and temperature (T) and salinity (S) in situ vertical profiles. At present, there are weekly and monthly mean 3D data for temperature, salinity, Ug, Vg and sea surface height data, and the spatial resolution is 1/4°. The vertical direction from 0 to 5500 m is divided into 33 layers (0, 10, 20, 30, 50, 75, 100, 125, 150, 200, 250, 300, 400, 500, 600, 700, 800, 900, 1000, 1100, 1200,1300, 1400, 1500, 1750, 2000, 2500, 3000, 3500, 4000, 4500, 5000, and 5500 m). The commercial data are very similar to our on-site observations, and the RMS error is lower than the climatic field.

## Results

### Time series of intermediate water

**Figure 2:**
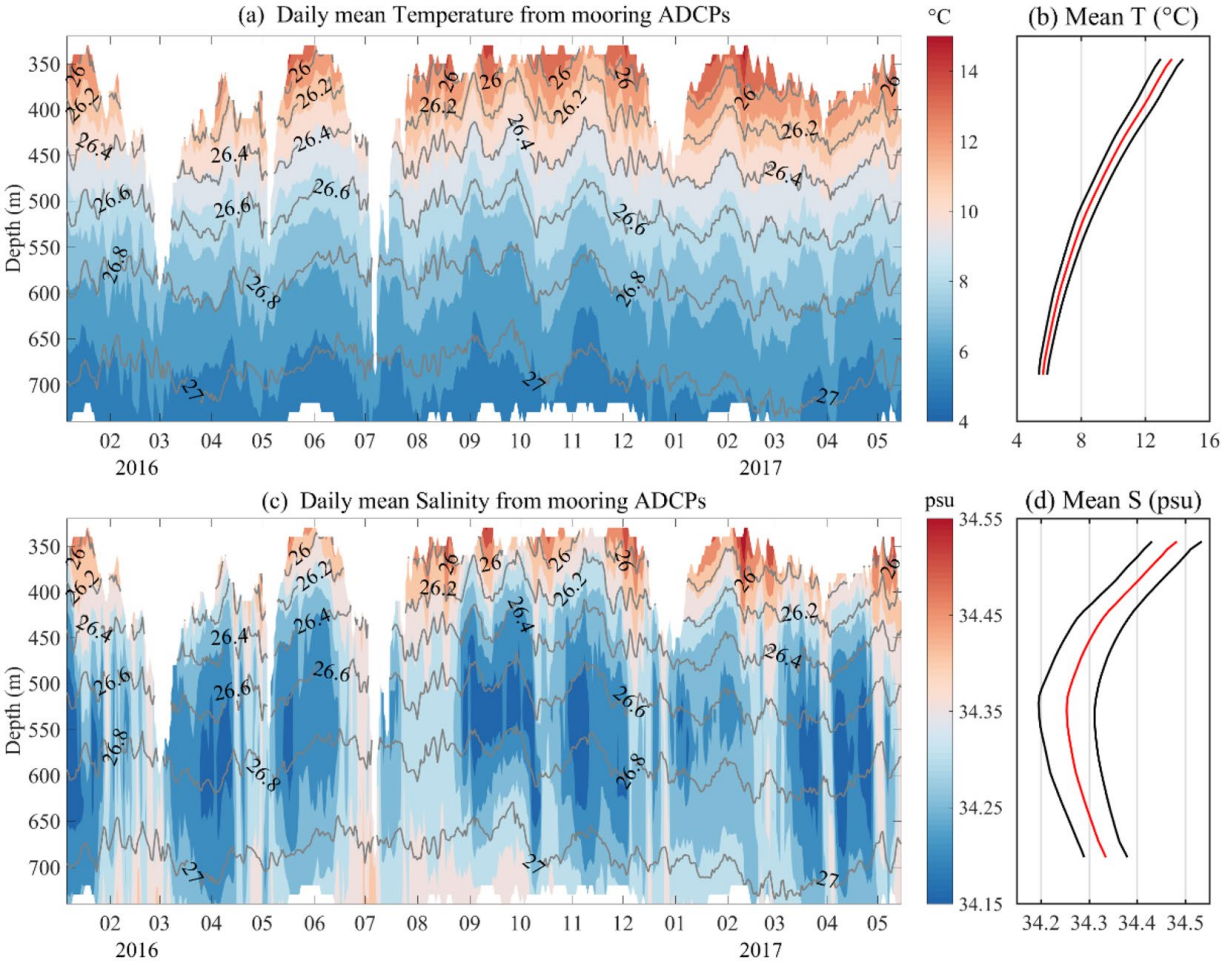
Time series of temperature and salinity from mooring observation. The daily mean of the CTD measurements of the temperature and salinity at depths from 350 to 800 m during the period from Jan 2016 to April 2017 in (**a**,**c**), respectively. Gray in (**a**,**c**) indicates the potential density calculated from temperature, salinity and depth data measured by the CTDs. The red lines in (**b**,**d**) indicate the means, and the black lines in (**b**,**d**) indicate the deviations of the temperature and salinity, respectively.

Because the main float of the mooring system was affected by the current, its depth changes greatly, which led to deviations of the entire mooring system. Therefore, all the instruments and equipment designed for use at a predetermined depth were basically in a fluctuating state. The daily average data in Fig. 2 shows large fluctuations for a maximum floating depth of 300 m. The results for the current show the velocity structure and variation characteristics of the Kuroshio (Fig. S2). The most significant variation in the Kuroshio east of Taiwan is expressed as intraseasonal variations with periods of ~ 85 d, and these variations are mainly modulated by mesoscale eddies propagating westward from the STCC, mainly including Kuroshio velocity, transport and main axis migration, et al.^3,21,26^. The relationship between significant intraseasonal variations in the Kuroshio and IW is one focus of this study. The minimum salinity ($S_{min}$) at the core of the intermediate water, shown in Fig. 2c, is found mainly in the potential density range of 26.6–26.8 $\sigma_{\theta }$, where $S_{min}$ is approximately 34.15 psu and the depth is approximately 600 m, with corresponding IW temperature range from 7 to 8 °C showed in Fig. 2a. We also found that $S_{min}$ at the core showed discontinuous variability; for example, $S_{min}$ at the core was approximately 600 m during the period March–April 2016, and $S_{min}$ was approximately 550 m by September 2016. There are 7 results for $S_{min}$ at the core in the observation period, at the same time, we found in Fig. 2 that there is some upward increase in isothermal and the isopycnal during these low-salt events, while there is a downward trend in isothermal and the isopycnal except during these low-salt events. According to the total measurement time, and an intraseasonal variation period of approximately 70–80 days was estimated. The mean salinity of each layer is shown in Fig. 2d, although the overall standard deviation was relatively small, the variance was larger at 500–550 m than in the other layers, indicating a relatively large variation in salinity in the middle layer. Of course, the standard deviation of temperature measurements decreases with increasing depth showed in Fig. 2b.

### T-S characteristics of intermediate water

**Figure 3:**
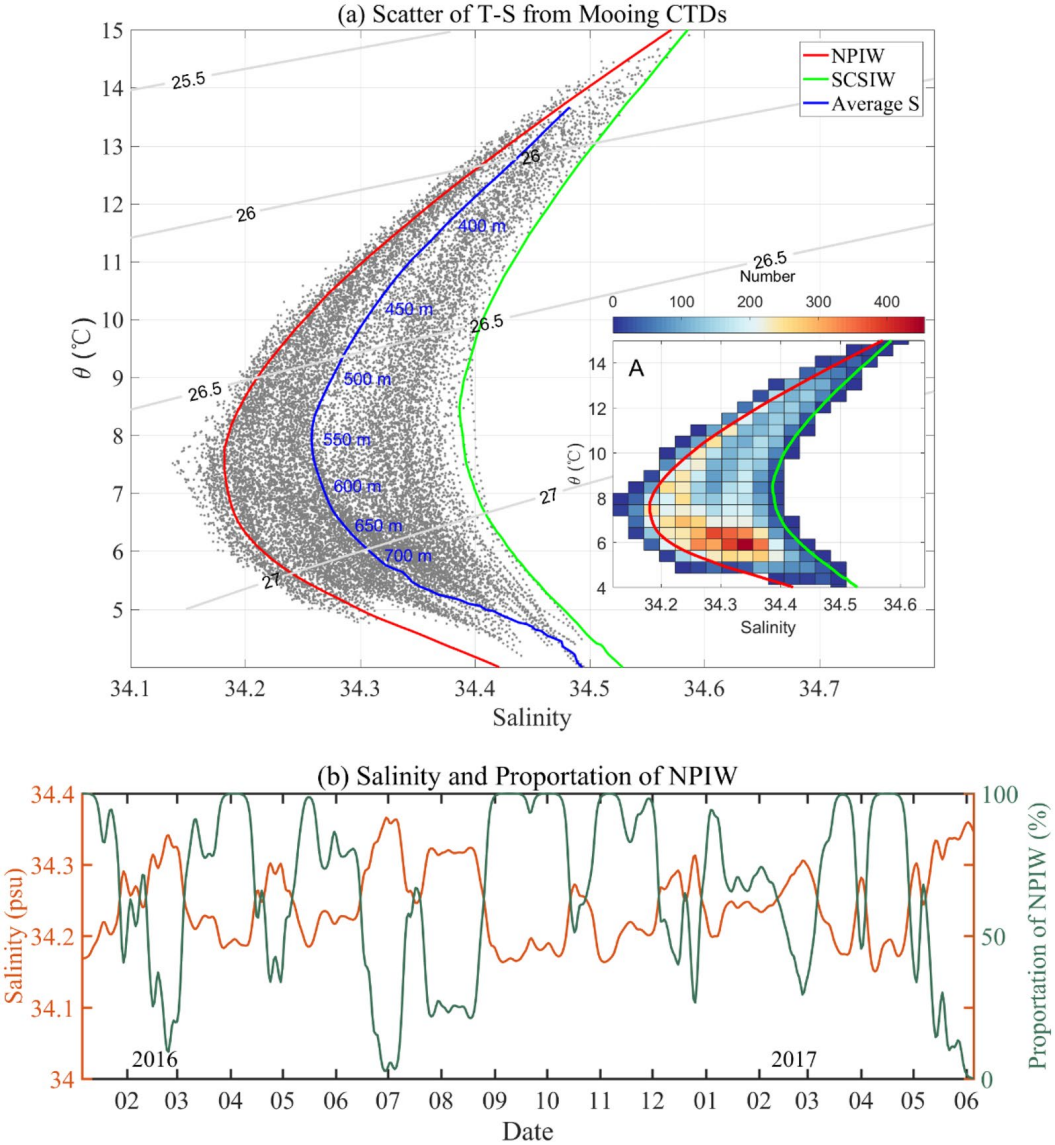
T-S scatter plot and time series of minimum salinity**. (a)** T-S scatter diagram obtained from moored CTD measurements. The gray points are CTD data, the red and green curves are the average temperature and salinity curves obtained from historical Argo data in the range of box A1 in Fig. 1a, and box A2 in Fig. 1a. Panel A in Fig. 3a represents the distribution of observation number (gray points in Fig. 3a) in each grid. (**b**) The time series of minimum salinity (orange curve) and the proportion of NPIW (green curve).

To more clearly analyze the characteristics of IW east of Taiwan, we drew a T-S scatter plot of data obtained from the moored CTDs (Fig. 3a). For comparison, historical data from the Argo international project for average temperature and salinity east of Taiwan but away from the Kuroshio area (box A1 in Fig. 1a) and the South China Sea area (box A2 in Fig. 1a) represent NPIW and SCSIW, respectively. The $S_{min}$ values of NPIW and SCSIW are 34.18 psu and 34.39 psu, respectively.

The main characteristics of IW in the water east of Taiwan are as follows: $S_{min}$ varies from 34.15 to 34.4 psu, corresponding to a temperature change in the range of 7 to 8 °C and a potential density variation of 26.6–26.8 $\sigma_{\theta }$ for $S_{min}$, respectively. The salinity distribution near the characteristic salinity value of NPIW is more concentration than SCSIW, also demonstrated form panel A in Fig. 3a (near the red curve), indicating that the overall characteristics of the water mass are closer to those of NPIW during the observations. Numerical results show only two moments in time when $S_{min}$ exceeded 34.39 psu in Fig. 3a, indicating that observations of IW with typical SCSIW characteristics are relatively infrequent. That is, most of the time, IW in the water east of Taiwan resembles a mixture of NPIW and SCSIW. Chern and Wang^16^ proposed that the SCSIW could not flow directly to eastern Taiwan due to blocking by the Green Islands in southeastern Taiwan, because the ridge between Taiwan and Green Island is shallow than 500 m. Mensah et al.^6^ report that NPIW and SCSIW could mix at the relatively southern location of the Luzon Strait, and the Kuroshio carried this water mass to the east of Taiwan. Therefore, we can obtain the typical average characteristics of this water mass, and the average ${\uptheta }$-S curves are plotted in Fig. 3a (blue curve). $S_{min}$ and the corresponding temperature of this water mass were 34.28 psu and 7.5 °C, respectively. The core was located at 550 m, which corresponded to a depth between the $S_{min}$ core depths of 500 m for SCSIW and 600 m for NPIW.

In this paper, a more widely used definition of KIW is adopted, which is considered to be mainly mixing between NPIW and SCSIW. To explore the ratio of the two water masses east of Taiwan, the concentration calculation equation was applied. First, salinity values of 34.19 psu and 34.39 psu were taken to characterize low-salt core of NPIW and SCSIW, respectively, according to the red and green curves in Fig. 3a, and the $S_{min}$ of each profile measured by the CTDs was taken to be the value of the two mixtures. Then, these three values were introduced into the concentration equation to calculate the mixing ratio. Figure 3b shows that the proportion of NPIW in KIW was relatively high, and 70% of the time, the NPIW mixing ratio exceeded 60%. There were approximately 7 moments in time when the proportion of NPIW reached 100%, which meant that there was basically only NPIW in the area east of Taiwan; these moments were evenly distributed during the observation period. The longest duration lasted for approximately one month in September 2016. Meanwhile, there were 4 moments when the proportion of NPIW was very small, such as March 2016, July 2016, August 2016, and June 2017. The proportion of NPIW in July 2016 and June 2017 was almost zero, indicating that there was basically no NPIW east of Taiwan, and SCSIW was predominant.

The results of previous studies basically on cruise sections data, but can only provide characteristics at certain moments.Therefore, SCSIW was found to exist east of Taiwan^17,18^, while others were not found in their study^16^. We used continuous salinity monitoring data to demonstrate that dynamic changes in salinity in the region may be responsible for the observed differences in salinity characteristics in these studies.

### Intraseasonal variability of intermediate water

**Figure 4:**
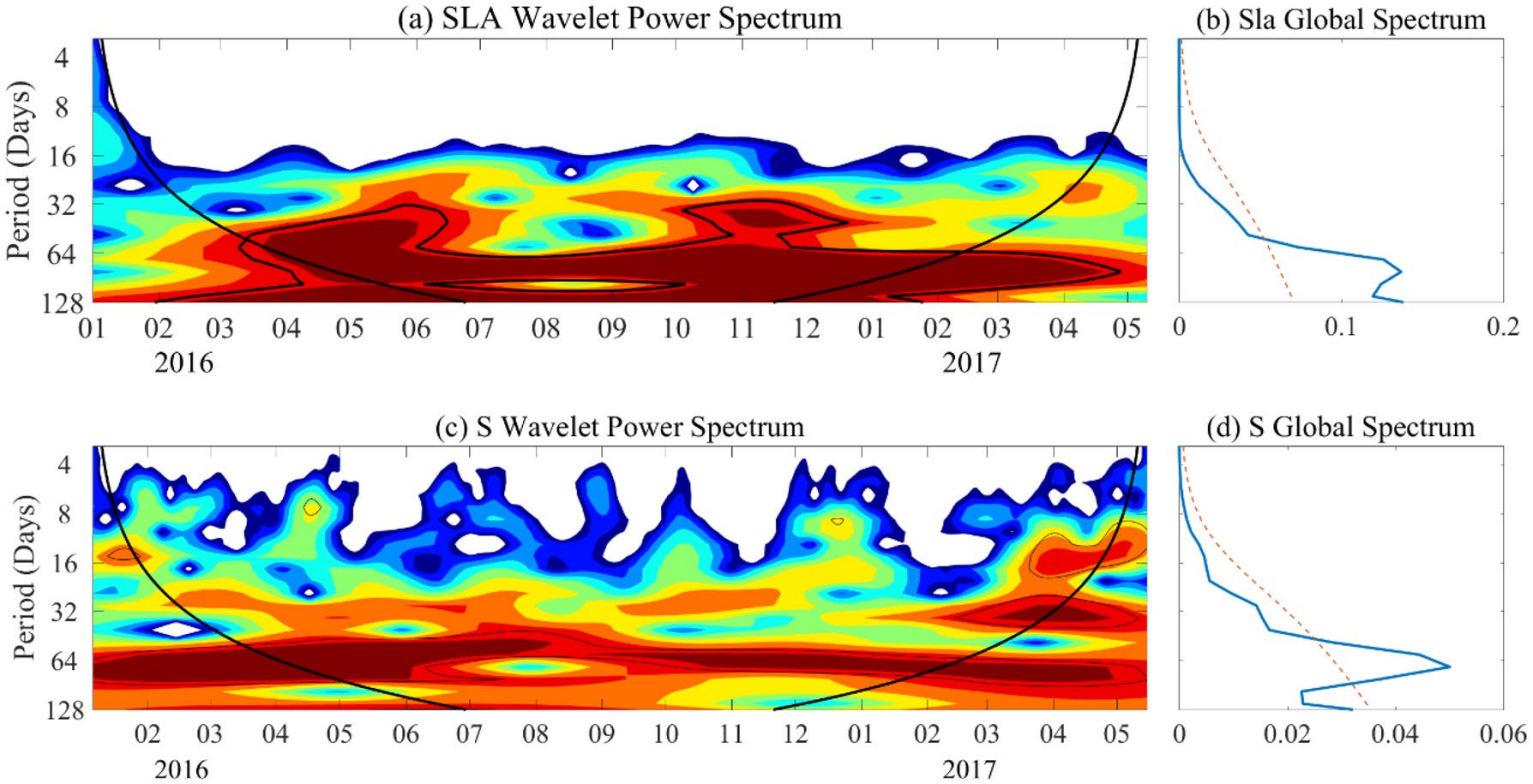
Wavelet power spectrum of SLA and salinity. (**a**) The SLA Wavelet power Spectrum at mooring site 122° E, 23° N from AVISO. The thick black contours represent 95% significance level. (**b**) The corresponding global power spectrum of SLA in Fig. 4a, the red dashed line denotes the 95% significance level. (**c**,**d**) The same as Figs. 4a and 4b but for salinity averaged between 26.4 and 27.0 σθ.

Figure 4c,d show the wavelet analysis for the salinity averaged over 26.4–27.0 $\sigma_{\theta }$, capturing the characteristics of intermediate salinity variability. The wavelet power spectrum showing significant power peaks with intraseasonal variability (ISV) periods of 70–80 days. The ISV signals showed very strong characteristics except in August to September 2016, although the signals before April, 2016 and after March, 2017 did not pass the 95% significant level, but they still had larger power. Through a reverse calculation of salinity based on an empirical temperature-salinity formula, Mensah et al.^6^ reported that the intraseasonal period of IW east of Taiwan was ~ 100 days. Compared with the result of Mensah et al.^6^, the intraseasonal signal of IW obtained from directly measured salinity data in this study may be more realistic reflection of the variation characteristics of the water mass. Of course, it is also possible that the difference in results is due to the timing of the two observations.

**Figure 5:**
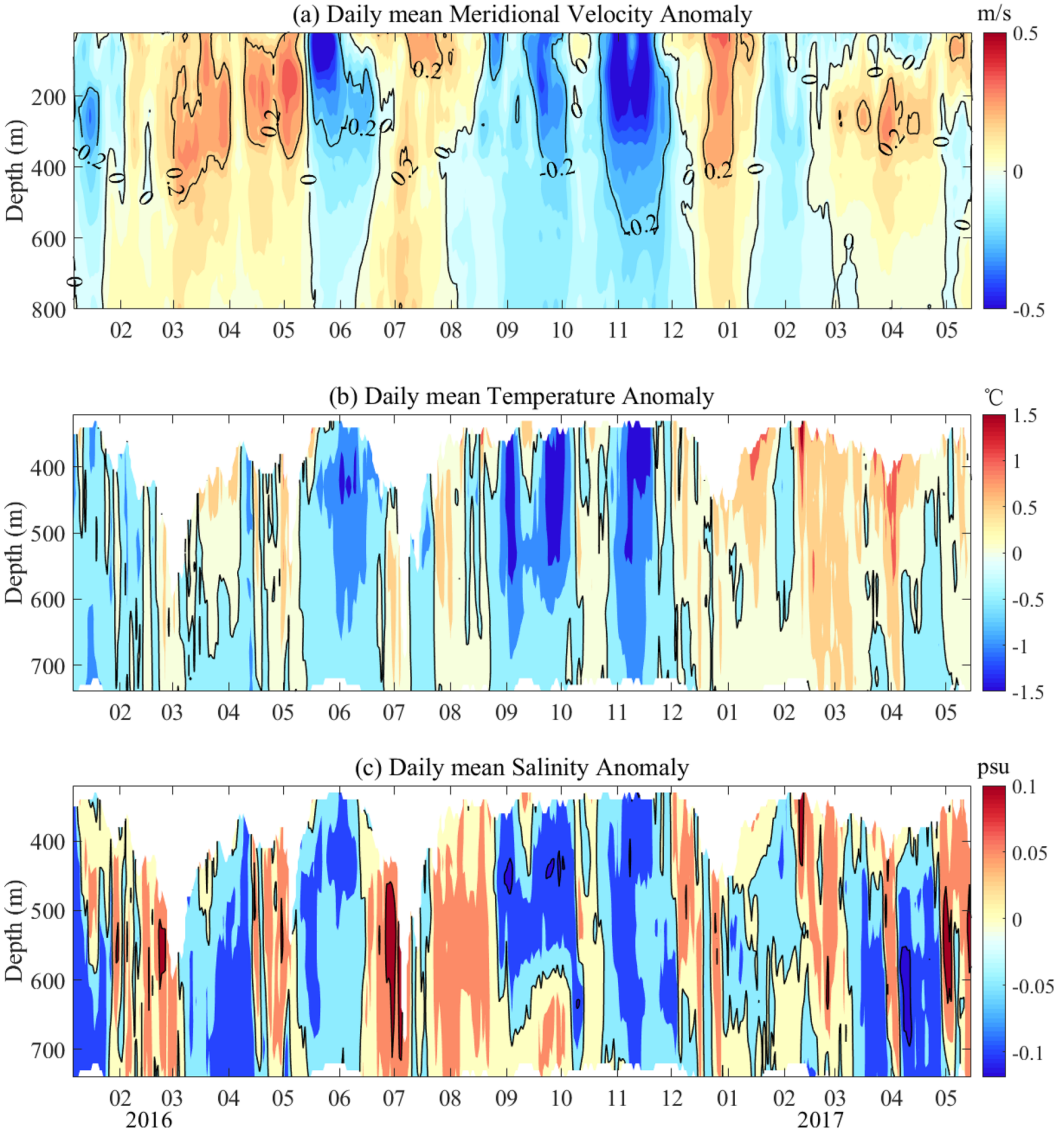
Anomaly of meridional velocity, temperature and salinity. (**a**) meridional velocity anomaly from 0 to 800 m; (**b**) salinity anomaly from 400 to 800 m; (**c**) temperature anomaly from 400 to 800 m. The meridional velocity, temperature and salinity anomalies were subtracted from the average values at each level over the entire observation time. The black contours indicate the zero-line values.

To better understand the intraseasonal variability, the meridional velocity anomaly, temperature and salinity anomaly were calculated by subtracting the average value during the observation of each layer from the average daily mooring data displayed in Fig. 5a–c. The meridional velocity anomalies are basically consistent and banded in the 0–800 m range, and temperature and salinity anomalies also exhibit synchronization in the 400–800 m range. The alternating band structures of positive and negative shapes are clearly shown in the anomalies temperature and salinity graphs, also indicating an intraseasonal signal of approximately 3 months. The maximum negative and positive salinity anomalies were − 0.12 psu and 0.1 psu, and the maximum negative and positive temperature anomalies were − 1.5 °C and 2 °C, respectively. During the observation period, there were 6 negative salinity anomalies in 17 months, March–April, June, September–October, and November–December in 2016 and January–February and April–May in 2017; the positive anomalies occurred during the other observation times. Meanwhile, the temperature and salinity anomalies were consistent and showed synchronous changes. The distribution of the integrated current anomalies showed that at most moments, positive meridional velocity anomalies corresponded to positive anomalies of temperature and salinity, while negative meridional velocity anomalies corresponded to negative anomalies of temperature and salinity. The relatively consistent variation in current, temperature and salinity suggests that all three parameters may be influenced by the same factor.

## Discussion

### Intraseasonal variability of IW caused by mesoscale eddies

**Figure 6:**
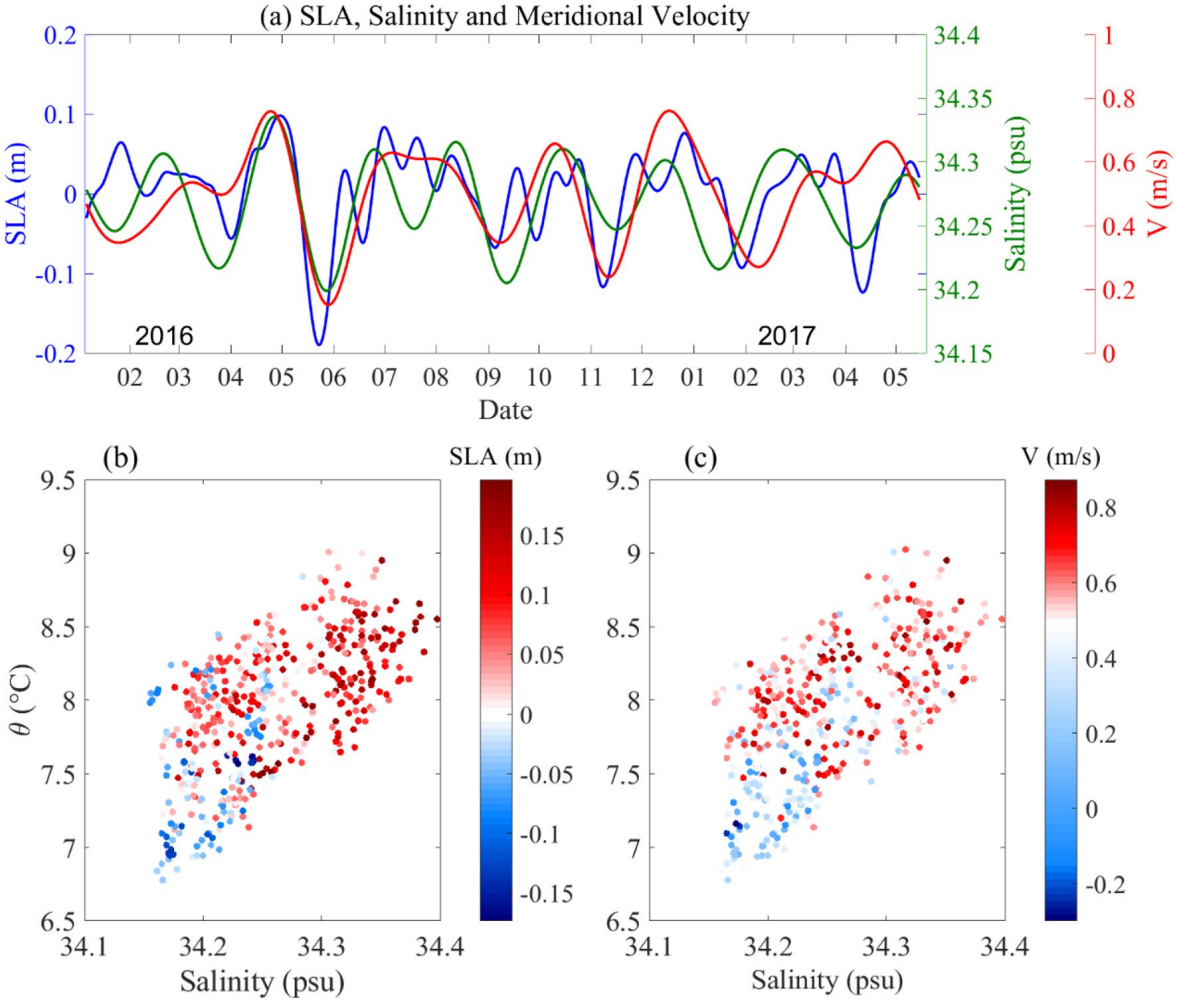
The relationship between the SLA, current and salinity in the intermediate layer. (**a**) Plot of 20–100 day bandpass-filtered time series of SLAs (blue curve), minimum salinity (green curve) and average velocity in the 0–400 m range (red curve). The salinity and velocity data are shown in (**b**) a T-S scatter diagram where the color indicates SLAs and (**c**) a T-S scatter diagram where the color indicates velocity.

The wavelet power spectrum of the SLAs located at mooring site in Fig. 4a,b showed the strong intraseasonal variability with periods ~ 80 days throughout the observation time, indicating a possible relationship between mesoscale eddies and IW. Also, we checked the local wind stress has a period of ~ 15 days (figure not shown) is inconsistent with the ~ 80 days variability of the IW. This maybe indicates that the local wind is not a direct cause of the intraseasonal variability of the IW. To identify the relationship between mesoscale eddies, Kuroshio velocity and IW, we plotted the time series of the SLAs; the salinity averages were between 26.4 and 27.0 $\sigma_{\theta }$ and the Kuroshio velocity averages between 0 and 400 m are shown in Fig. 6a. The results showed that they exhibited significantly consistent variations, with correlation coefficients of 0.63 and 0.52 between the SLA and Kuroshio velocity and between the SLA and salinity, respectively. Figure 6b,c show scatter plots of the measured potential temperature against salinity, composite with the SLAs and averaged V, respectively. Most of the fresher (saltier) water corresponded to negative (positive) SLAs displayed in Fig. 6b. Additionally, stronger currents carry saltier water, while only weak currents can carry lower temperature and fresher water; this is especially significant in the case of the southward-flowing current shown in Fig. 6c. The above results indicate that changes in the synchronization of temperature, salinity, and current were highly correlated with SLAs. Previous studies argued that the pronounced ISV at eastern Taiwan was mainly caused by western propagating mesoscale eddies^3,22,23,27^, The mesoscale eddy induced the meridional velocity, transport and main axis of Kuroshio show strong variations, Ren et al.^26^ had been reported the typical westward propagation characteristics of mesoscale eddies along the 23° N section east of Taiwan during Jan 2016 to June 2017. Combined variations in V, salinity and temperature anomalies, and their corresponding relationships with SLAs, we conclude that the positive (negative) SLAs caused by westward propagation of anticyclonic (cyclonic) eddies from the STCC increases (decreases) the speed of the Kuroshio while increasing (decreasing) temperature and salinity at approximately 400–600 m. In other words, changes in both temperature and salinity in the intermediate layer and the Kuroshio can be traced to mesoscale eddies western propagating from the STCC.

**Figure 7:**
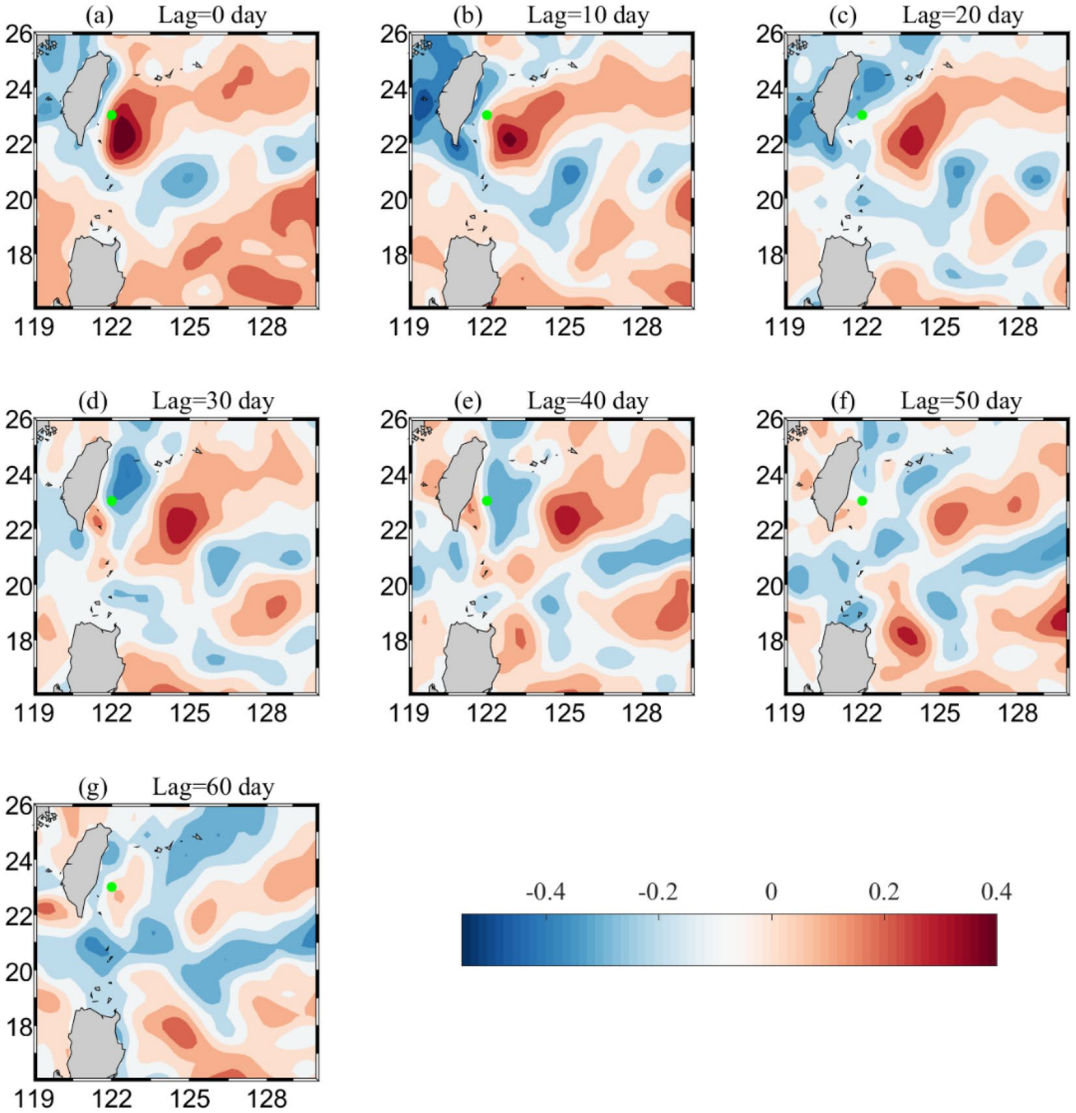
Maps of correlation coefficients between SLAs and minimum salinity of IW. Salinity lagged SLA by (**a**) 0 days, (**b**) 10 days, (**c**) 20 days, (**d**) 30 days, (**e**) 40 days, (**f**) 50 days and (**g**) 60 days. Color shading indicates the value of the correlation coefficient, with red (blue) indicating a positive (negative) correlation. The location of the mooring site is indicated by green dot. Figures were plotted using MATLAB R2016b (http://www.mathworks.com/).

To further analyze the time-scale relationship between mesoscale eddies and IW, we constructed a lag correlation coefficient diagram of salinity in the range 26.4–27.0 $\sigma_{\theta }$ and SLAs within the region 119–130° E/18–25° N (Fig. 7). Figure 7a–g show that salinity lagged behind SLAs from 0 to 60 days with an interval of 10 days. East of Taiwan, there was an area with a positive correlation coefficient (red) that gradually moved westward with time from the 60th day onward, and the correlation coefficient increased with its maximum increasing from 0.3 on the 60th day to 0.5 on the 0th day. There was a significant area with a positive correlation coefficient located at 125° E/22° N with a 60-day lag relative to the mooring site, while at the highest area located at 122° E/22.5° N with 0 lag time; as a result, the estimated westward propagation velocities of the mesoscale eddies were approximately 10 cm/s. As a comparison, Tsai et al.^27^ used PIES data to conclude that the westward speed of an eddy near Taiwan was in the range of 11–24 cm/s. Also, the westward propagation speed of mesoscale eddies of first-mode baroclinic Rossby waves near 20° N was approximately 8 cm/s^2^.

### Possible mechanism of IW variations

Measurements at the mooring site, a single point, are not enough to reflect the movement of salinity and the current-water mass relationship. Therefore, a wider range of Global Ocean Multi Observation Products (GOOP) data from global ocean observation was used to analyze this behavior. First, we examined the consistency between data from the moored CTDs and GOOP. The time series chart of GOOP salinity data is shown in Fig. S3 and compared to salinity data from the moored system in Fig. 2c. The salinity characteristics corresponded better at several moments, e.g., the low salinity characteristics in January, April, June, September, and November 2016 and January and May 2017, and the relatively high salinity characteristics in the period from July to August 2016, than at other times. Although the agreement was not complete, it is enough to indicate that the variation characteristics of salinity from GOOP are similar to those from the in situ measurements.

**Figure 8:**
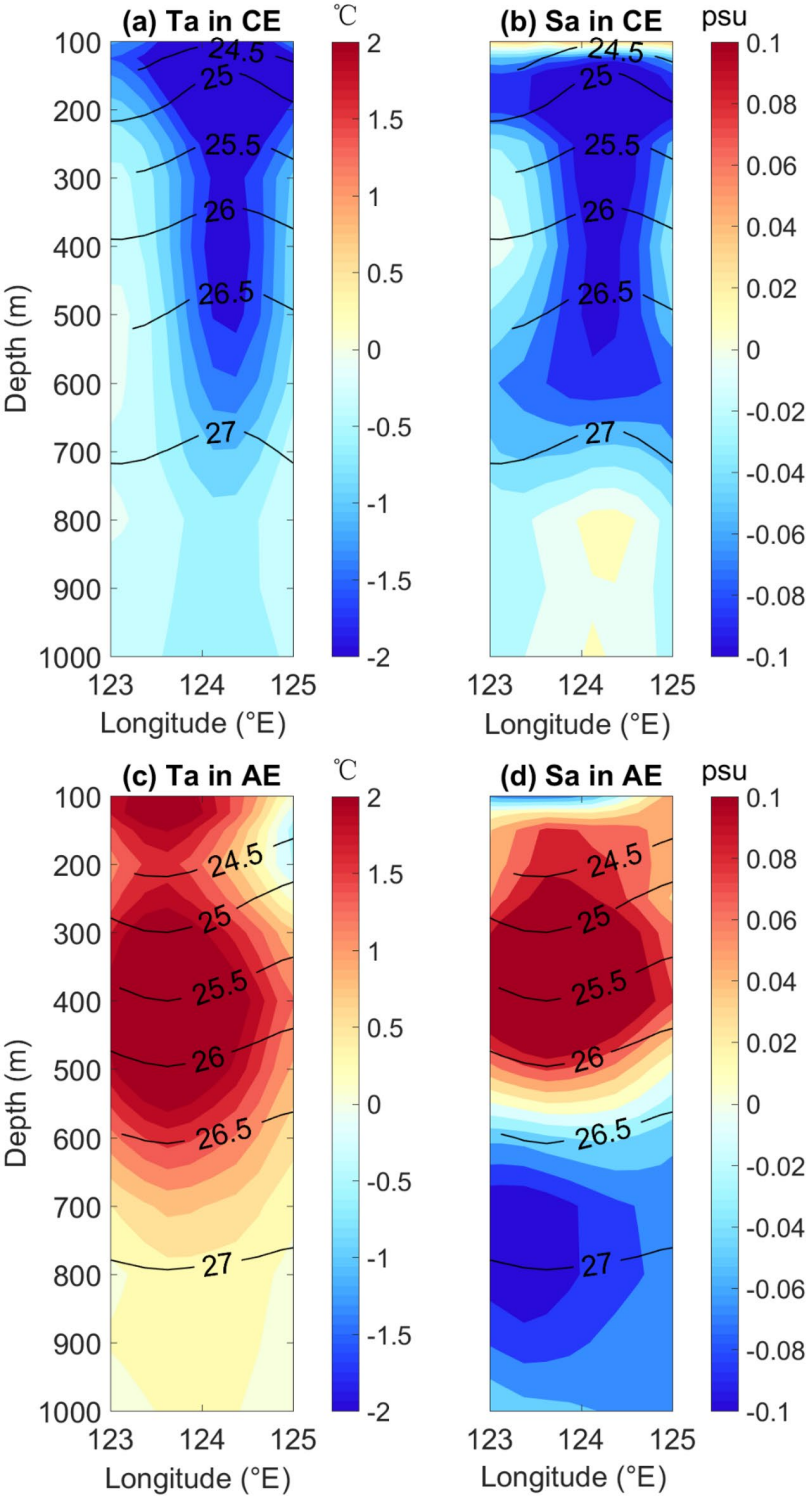
The structure of anticyclonic and cyclonic eddies. Temperature anomaly (**a**) and salinity anomaly (**b**) of cyclonic eddies. Colors indicate temperature and salinity anomalies, and contour lines indicate potential density (**c**,**d**) for anticyclonic eddies.

Actually, the salinity at the intermediate layer was analyzed according to horizontal and vertical movement. First, we constructed the vertical structure of the anticyclonic and cyclonic eddy times to discuss IW movement in the vertical direction. Figure 8a–d show the anomalies temperature and salinity distributions from 0 to 1000 m along the center of cyclonic and anticyclonic eddies, respectively. In anticyclonic (cyclonic) eddies, isothermal and isosalinity lines showed obvious concave (convex) structures corresponding to vertical downward (upward) movement of the water mass at the center, corresponding the positive (negative) temperature anomalies. However, the structure was different for temperature and salinity because the water mass east of Taiwan was divided into subsurface high-salinity water at 100–200 m and intermediate fresher water at 400–600 m. The downward movement of water at the centers of the anticyclonic eddies caused subsurface high-salinity water to flow downward and mix with relatively fresh water in the intermediate layer and cause positive anomalies above 600 m; as fresher intermediate water moved downward, it caused negative anomalies in the deep layer below 600 m. And inside the cyclonic eddies the salinity structure displayed negative anomalies above 600 m and positive anomalies below 600 m in cyclonic eddies, as shown in Fig. 8d. With the upward movement of the water mass, fresher intermediate water mixed with high salinity water and caused negative salinity anomalies in upper intermediate layers, while the high salinity of the deep water moving upward caused positive salinity anomalies in the deep layer. From the above analysis of salinity movement in the vertical direction, the results show that temperature and salinity increased (decreased) in intermediate layers due to vertical movement of water by anticyclonic (cyclonic) eddies. According to the existing definition, the salinity of NPIW is range between 34.1 and 34.3 psu, so if the vertical motion is relatively small and mixing is weakening by weak anticyclonic eddies, even if the salinity increases in intermediate layer, but the water mass may still exhibit the characteristics of NPIW. Combined with the above analysis, the vertical motion of the water masses caused by the mesoscale eddies is one of the reasons for the temperature and salinity changes inside the eddies. However, it can also be considered that the salinity and temperature changes does not mean that the water mass will definitely change from NPIW to SCSIW, and it may also need to be considered from the perspective of horizontal transport mixing of different water masses caused by mesoscale eddies.

**Figure 9:**
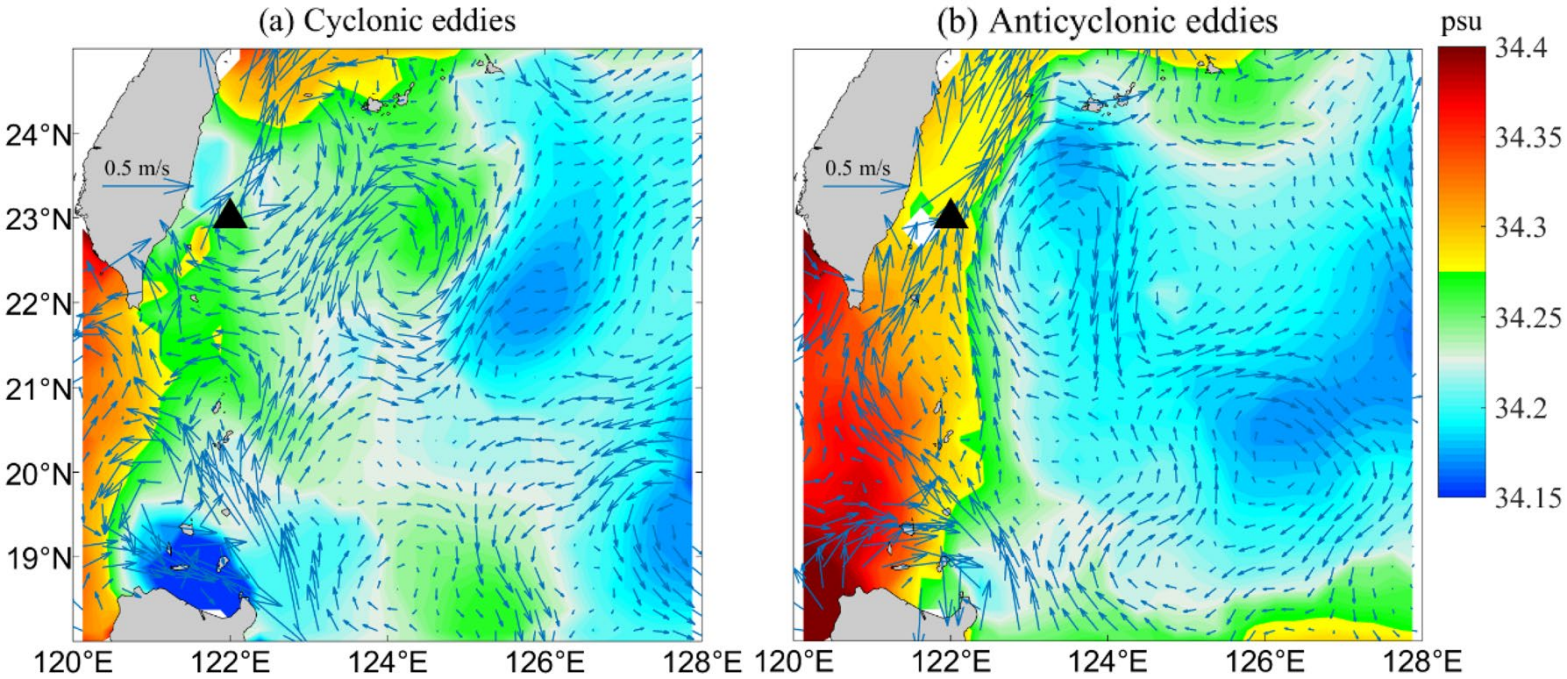
Relationship between the circulation and water mass off the eastern Taiwan. (**a**) Composite map of salinity (colors) and current (black arrows) averaged between 26.4 and 26.8 $\sigma_{\theta }$ east of Taiwan for cyclonic eddies and (**b**) a composite map of anticyclonic eddies. The black triangle is the mooring site. Figures were plotted using MATLAB R2016b (http://www.mathworks.com/).

The above analysis only considers vertical movement of water induced by mesoscale eddies. Next, we discuss horizontal movement of water combined with currents in intermediate layers. Figure 9a,b show composite circulation and salinity during cyclonic and anticyclonic eddy periods with the velocity average in the range of 26.4–26.8 $\sigma_{\theta }$ east of Taiwan.

Figure 9a,b show different salinity distribution characteristics in the South China Sea (SCS) and North Western Pacific (NWP) when the Kuroshio interaction with the mesoscale eddies. One of the obvious features presented in the Fig. 9 show that there is a higher salinity gradient from west to east during the anticyclonic eddies period, also most of the Luzon Strait is occupied by high salinity SCS water. When cyclonic eddies impinge on the Kuroshio (Fig. 9a), the current is weakened by cyclonic eddies, and relatively fresher water surrounds the intermediate layer east of Taiwan. Additionally, Fig. 9a shows that little water is carried out of the SCS by the Kuroshio, which means that NPIW more easily dominates water east of Taiwan and maintains lower salt characteristics. In contrast, during anticyclonic interactions with the Kuroshio, the current is strengthened to the north, and the field of salinity is significantly higher in the intermediate layer along Luzon Island to the Taiwan coast (Fig. 9b).

In fact, we speculate that the increase in IW salinity may be associated with the path of Kuroshio intrusion into the SCS caused by the anticyclonic eddies. When the presence of the anticyclonic eddies in the east of Luzon Strait, forcing the Kuroshio to bend toward the SCS, will lead to the Kuroshio intrusion into the South China Sea to form a loop structure, can reach a depth of about 1000 m^28–30^. Therefore, the horizontal flow velocity and vertical influence depth of the Kuroshio will be enhanced, and may be more SCS water will be brought out from the SCS to the area east of Taiwan, thus increasing the salinity of the intermediate layer. During the cyclonic eddies, the northward transport capacity weakens with the Kuroshio weakens. Qu et al.^31^ and Wang et al.^32^ report that most of the intermediate water layer flows southward east of Luzon, even the intermediate water flow occasionally reverses to a southward course at southeast of Taiwan^6^, this also can be find in Fig. S2. Therefore, it is may be relative difficult for the SCS waters out of the Luzon Strait to be transported to the north by weaken Kuroshio, the southward flow on the west font of the cyclonic eddies restrains the northward transport and is able to carry the NPIW on the east side to the east of Taiwan.

Therefore, the results reveal that horizontal movement of IW carried by current is possible as follows. Anticyclonic eddies strengthened the Kuroshio and benefitted SCSIW flowing through the Luzon Strait, leading to an increase in salinity east of Taiwan. While cyclonic eddies weakened the Kuroshio and reduced SCSIW outflow from the Strait, this was conducive to relatively low-salt NPIW occupying the area east of Taiwan. Evaluating the influence of the IW east of Taiwan by the way and path of the Kuroshio intrusion into the SCS is the next step to be explored.

## Summary

This study utilizes 17 months of long-term, continuous and synchronous measurements of temperature, salinity and current data from a mooring site located at 122° E/23° N east of Taiwan. The IW characteristics in eastern Taiwan were revealed as follows: $S_{min}$ varied from 34.15 to 34.4 psu, corresponding to a temperature variation in the range from 7 to 8 °C and a potential density variation of 26.6–26.8 $\sigma_{\theta }$ for $S_{min}$. We observed pronounced intraseasonal variations in IW with periods of ~ 80 days.

For the first time, we prove that intraseasonal variability (ISV) of IW are caused by mesoscale eddies propagating westward from the STCC. The mesoscale eddies not only influenced the Kuroshio but also brought intraseasonal signals to the water mass of the intermediate layer. The correlation coefficients between SLAs and current and between SLAs and salinity in the intermediate layer were 0.63 and 0.52, respectively. Results showed that positive (negative) SLAs, caused by the westward propagation of anticyclonic (cyclonic) eddies from the STCC, increased (decreased) the speed of the Kuroshio, also increase (decrease) the temperature and salinity in the 400–600 m in east of Taiwan. By using the map of correlations between SLAs and $S_{min}$, the westward propagation speed of the mesoscale eddies was estimated to be approximately 10 cm/s.

Combined the GOOP data, the movement of IW are discussed. The vertical movement of IW showed that the temperature and salinity increased (decreased) in the intermediate layer due to the vertical movement of water by anticyclonic (cyclonic) eddies. Meanwhile, during the horizontal movement of the water mass, anticyclonic eddies strengthened the Kuroshio and benefitted SCSIW flowing through the Luzon Strait, leading to an increase in salinity east of Taiwan. While the cyclonic eddies weakened the Kuroshio and reduced SCSIW outflow from the Strait, conditions were conducive to relatively low-salt NPIW in the area east of Taiwan.

## Supplementary Information

Supplementary Information.
