# Supplementary material for: Effects of mesoscale eddies on intraseasonal variability of intermediate water east of Taiwan
Source: Sci Rep. 2022 Jun 2;12:9182. doi: 10.1038/s41598-022-13274-2 (PMC9163106; doi:10.1038/s41598-022-13274-2)
Supplement: Supplementary file 1 — Supplementary Information. [file 41598_2022_13274_MOESM1_ESM.docx]

Effects of Mesoscale Eddies on Intraseasonal Variability of Intermediate Water East of Taiwan

Qiang Ren^1,2^, Fei Yu^1,2,3^, Feng Nan^1,2,3^, Yuanlong Li^1,2,3^, Jianfeng Wang^1,2,3^, Yansong Liu^1,2,3^, Zifei Chen^1,2^

^1^Key Laboratory of Ocean Circulation and Waves, Institute of Oceanology, Chinese Academy of Sciences, Qingdao, China.

^2^Center for Ocean Mega-Science, Chinese Academy of Sciences, Qingdao 266071, China.

^3^Pilot National Laboratory for Marine Science and Technology (Qingdao), Qingdao 266071, China.

Corresponding author: Fei Yu (yuf@qdio.ac.cn)

**Contents of this file**

Figures S1 to S3

**Supplementary Figures**


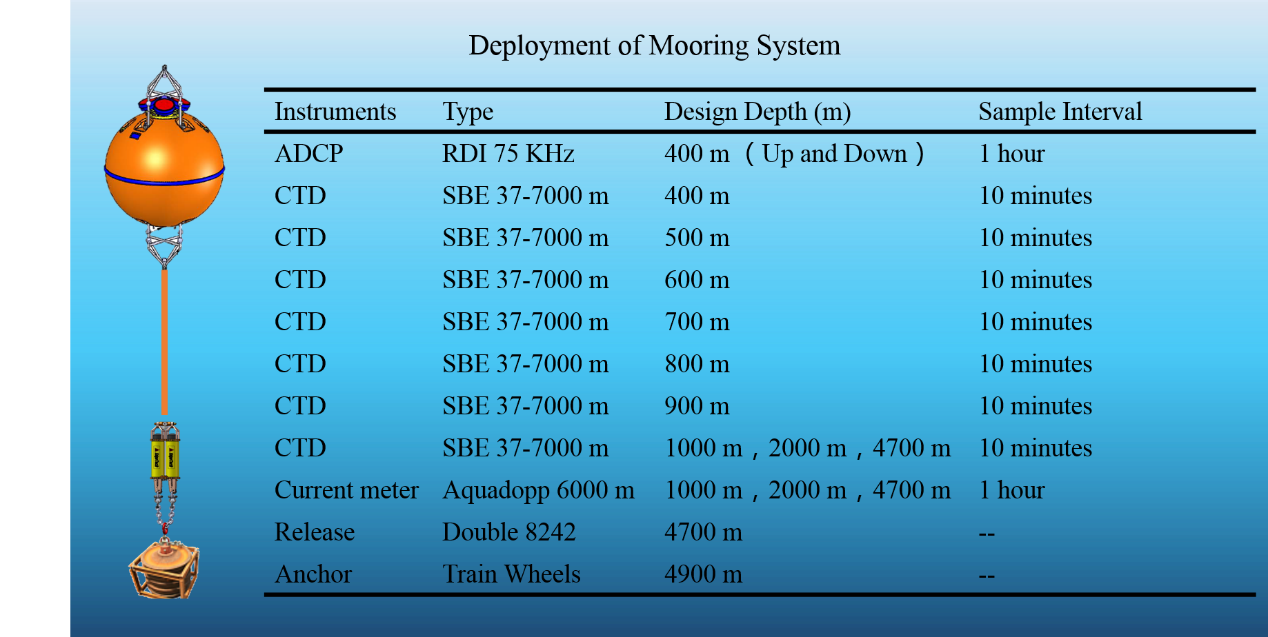


**Figure S1.** Simplified schematic diagram of the configuration and deployment of the subsurface mooring buoy system. The main float integration was two up-looking and down-looking 75 kHz RID ADCPs that were deployed at 400 m. CTDs were deployed from 400 to 1000 m at intervals of 100 m, and CTDs and current meters were deployed at 1000 m, 2000 m and 4700 m.


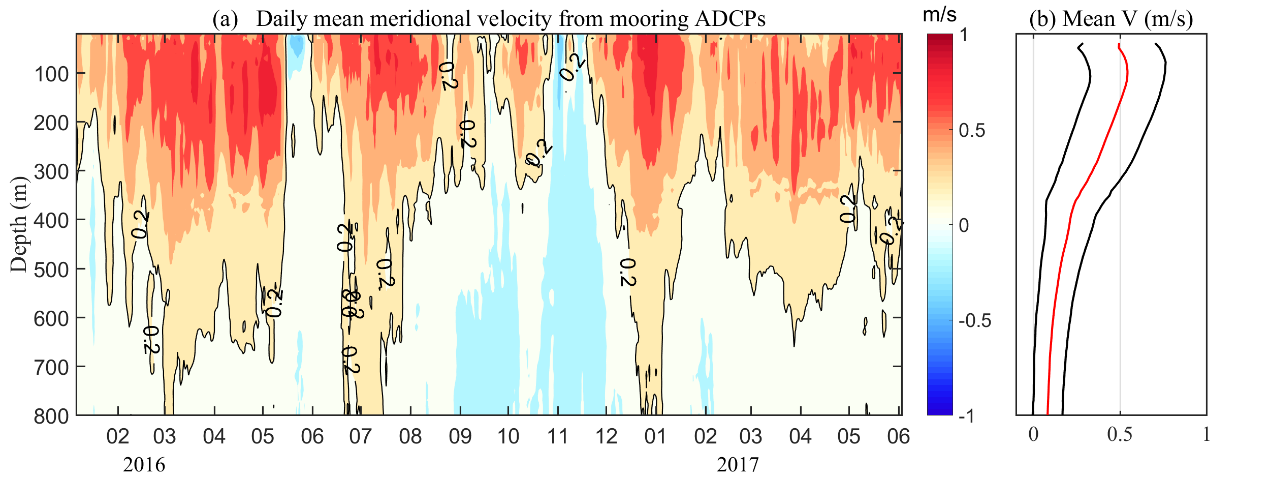


**Figure S2.** Time series of meridional velocity from mooring observation.


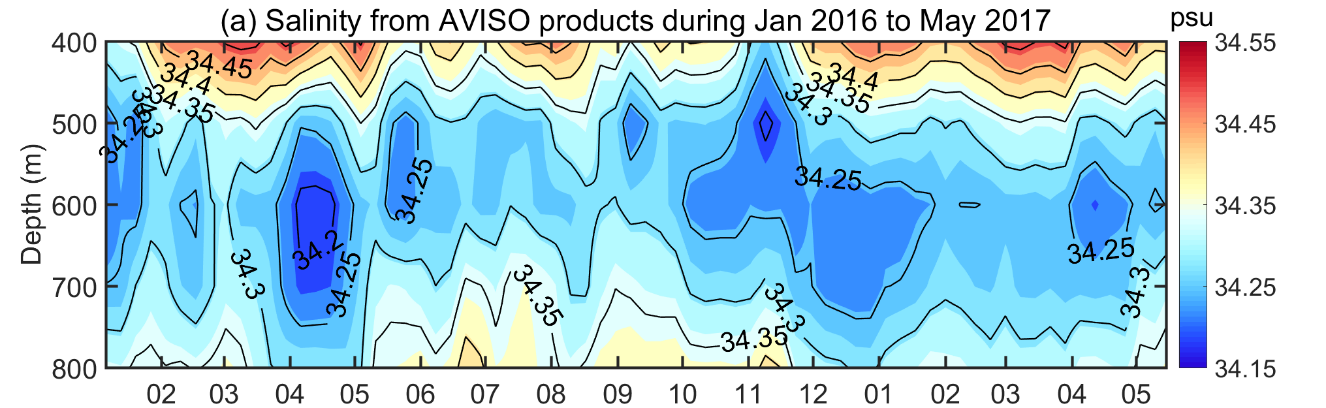


**Figure S3.** (**a**) Salinity time series from AVISO products from January 2016 to May 2017.
